# Efficiency of the Vitamin D Status Diagnosticator amongst Geriatric Patients with COVID-19

**DOI:** 10.3390/nu16060856

**Published:** 2024-03-15

**Authors:** Caroline Charonnat, Dolores Sanchez-Rodriguez, Spyridon N. Karras, Duygu Gezen-Ak, Erdinç Dursun, Cédric Annweiler

**Affiliations:** 1 Department of Geriatric Medicine and Memory Clinic, Research Center on Autonomy and Longevity CeRAL, University Hospital, 49000 Angers, France; 2 UNIV ANGERS, School of Medicine, Health Faculty, 49100 Angers, France; 3 Rehabilitation Research Group, Institut Hospital del Mar d’Investigacions Mèdiques (IMIM), 08003 Barcelona, Spain; 4 Geriatrics Department, Brugmann University Hospital, Université Libre de Bruxelles, 1020 Brussels, Belgium; 5 Geriatrics Department, Hospital Del Mar—Hospital de L’Esperança—Centre Fòrum, Parc de Salut Mar, 08029 Barcelona, Spain; 6 WHO Collaborating Centre for Public Health Aspects of Musculo-Skeletal Health and Ageing, Division of Public Health, Epidemiology and Health Economics, University of Liège, 4000 Liège, Belgium; 7 Laboratory of Biological Chemistry, Medical School, Aristotle University, 55535 Thessaloniki, Greece; 8 Brain and Neurodegenerative Disorders Research Laboratories, Department of Neuroscience, Institute of Neurological Sciences, Istanbul University-Cerrahpasa, 34098 Istanbul, Turkeyerdinc.dursun@iuc.edu.tr (E.D.); 9 UNIV ANGERS, UPRES EA 4638, 49045 Angers, France; 10 Gérontopôle Longévité Autonomie des Pays de la Loire, 44200 Nantes, France; 11 Department of Medical Biophysics, Robarts Research Institute, Schulich School of Medicine and Dentistry, the University of Western Ontario, London, ON N6A 5K8, Canada

**Keywords:** screening, vitamin D, vitamin D deficiency, older adults, COVID-19

## Abstract

The vitamin D status diagnosticator (VDSD), a 16-item tool, effectively identifies hypovitaminosis D in healthy older adults and can assist in determining the need for blood tests in this population. Assessing vitamin D levels is particularly crucial in the context of COVID-19. This study aimed to evaluate the VDSD’s effectiveness in pinpointing hypovitaminosis D in older adults affected by COVID-19. The research involved 102 unsupplemented geriatric inpatients consecutively admitted to the acute geriatric division of Angers University Hospital, France, with an average age of 85.0 ± 5.9 years (47.1% women). The physician-administered VDSD was conducted simultaneously with the measurement of serum 25-hydroxyvitamin D (25(OH)D). Hypovitaminosis D was defined as a serum 25(OH)D concentration of ≤75 nmol/L for vitamin D insufficiency and ≤50 nmol/L for vitamin D deficiency. Results revealed that 87 participants (85.3%) had vitamin D insufficiency and 63 (61.8%) had vitamin D deficiency. The VDSD accurately identified vitamin D deficiency with an area under the curve (AUC) of 0.81 and an odds ratio (OR) of 40. However, its accuracy in identifying vitamin D insufficiency was lower (AUC = 0.57). In conclusion, the 16-item VDSD, a concise questionnaire, effectively identifies vitamin D deficiency in geriatric patients with COVID-19. This tool can be valuable in guiding the decision to administer vitamin D supplementation during the early stages of COVID-19.

## 1. Introduction

Hypovitaminosis D, a common condition in older adults, is associated with various adverse health outcomes [1,2]. The immunomodulatory and anti-inflammatory properties of vitamin D [3] have led to its recent connection to an increased risk of COVID-19 [4], especially severe cases [5]. Studies have shown that individuals with lower levels of 25-hydroxyvitamin D (25(OH)D) during COVID-19 were more likely to require ventilation [6], have prolonged hospital stays [7], and face higher mortality rates from COVID-19 [8], including in intensive care units [9]. Conversely, normal vitamin D levels prior to SARS-CoV-2 infection have been associated with improved prognosis [10]. Similarly, supplementing with vitamin D early in COVID-19 has been shown to mitigate severity [11,12] and prevent mortality [13]. Identifying hypovitaminosis D in older adults at the onset of COVID-19 is thus crucial for timely supplementation and for determining the appropriate dosage. The challenge lies in the time required for blood tests and waiting for 25(OH)D results, which can take several hours or even days, risking delayed supplementation during the infection. We recently introduced the vitamin D status diagnosticator (VDSD), a 16-item questionnaire with a combinatorial non-linear algorithm that accurately identifies individuals with hypovitaminosis D without the need for a blood test. While its effectiveness has been demonstrated in healthy [14] and hospitalized [15] older adults, its utility in identifying hypovitaminosis D and guiding blood tests and supplementation in older adults with COVID-19 has not been explored. This analysis aims to evaluate the diagnostic efficacy of the VDSD tool in identifying hypovitaminosis D in geriatric patients with COVID-19.

## 2. Materials and Methods

### 2.1. Participants

We examined consecutively enrolled inpatients aged 75 years and older as part of the GERIA-COVID study. This longitudinal observational study was conducted in the geriatric acute care unit dedicated to COVID-19 patients at the University Hospital of Angers, France, during the initial and subsequent waves of the COVID-19 pandemic in the country (ClinicalTrials.gov NCT04560608). Data from the GERIA-COVID study were retrospectively gathered from patients’ records and a comprehensive description of the procedure is available elsewhere [16]. The inclusion criteria for both the GERIA-COVID study and the present analysis were as follows: (i) patients aged 75 years and above admitted to the geriatric acute care unit at the University Hospital of Angers, France, during the second wave of the pandemic (between November 2020 and March 2021); (ii) no objection from patients and/or their relatives regarding the utilization of anonymized clinical and biological data for research purposes; (iii) confirmed diagnosis of COVID-19 through RT-PCR and/or chest CT-scan; (iv) availability of data on circulating 25(OH)D concentration upon hospital admission; (v) availability of data on the vitamin D status diagnosticator (VDSD) results. The study adhered to the ethical standards outlined in the Declaration of Helsinki (1983). In this retrospective analysis of medical records, all data were fully anonymized. The ethics board of the University Hospital of Angers, France, granted approval for the study and waived the requirement for informed consent (2020/100). No participant or their relatives raised objections to the use of anonymized clinical and biological data for research purposes. The study protocol was also submitted to the French National Commission for Information Technology and Civil Liberties (CNIL; ar20-0087v0).

### 2.2. Vitamin D Status Diagnosticator

The development of the VDSD tool has been previously detailed [14]. In essence, the VDSD relies on a non-linear model of feed-forward artificial neural network (multilayer perceptron), constructed among community-dwelling older adults. All variables available in the database were incorporated into the model without any predefined hypothesis regarding their potential connection to vitamin D status. Their combination, rather than a direct link to vitamin D, was examined. Redundant variables were subsequently eliminated one by one based on their relative significance in the algorithm until the most effective minimum number of variables was determined. The final model is founded on 16 clinical items [14].

During this study, participants underwent a comprehensive clinical examination conducted by a physician to systematically gather the 16 items of the VDSD. These items include sex, age (in years), number of therapeutic classes used per day, body mass index (BMI, in kg/m^2^), use of walking aids, use of psychoactive drugs (such as benzodiazepines, anti-depressants, or neuroleptics), wearing glasses, sad mood, fear of falling, history of falls in the preceding year, cognitive disorders, undernutrition, polymorbidity, history of vertebral fractures, living alone, and use of anti-osteoporotic drugs (like bisphosphonates, strontium, or calcium). BMI was calculated based on anthropometric measurements, with undernutrition defined as a BMI below 22 kg/m^2^ [17]. Polymorbidity was identified as having more than three chronic diseases of indefinite duration or running a course with minimal change. A fall was defined as an event resulting in unintentional rest on the ground or at a lower level, not due to a major intrinsic event or overwhelming hazard, according to the French Society of Geriatrics and Gerontology (SFGG) and the French National Authority for Health [18]. The history of vertebral fractures was obtained from patient and relative interviews and medical records. The fear of falling was assessed using the standardized question “Are you afraid of falling?” [19]. The presence of cognitive disorders was noted, based on clinical expertise and/or history of dementia from medical records. Sad mood was evaluated using the question “Do you feel discouraged and sad?” from the 4-item geriatric depression scale [20]. Finally, without knowledge of the blood test results, we applied the previously published algorithm [14] to the VDSD items to identify individuals with hypovitaminosis D among those with COVID-19, differentiating between the 50 and 75 nmol/L thresholds [21,22].

### 2.3. Serum 25(OH)D Measure

Venous blood samples were obtained from individuals with COVID-19 during their resting state at the time of the VDSD assessment for the quantification of serum 25(OH)D concentration. The measurement of all serum 25(OH)D levels was conducted using chemiluminescent immunoassay (LIAISON XL, DiaSorin, Saluggia, Italy) in a singular laboratory situated at the University Hospital of Angers, France, adhering to the DEQAS scheme. In accordance with prior research, two distinct threshold values were sequentially applied to define hypovitaminosis D: vitamin D insufficiency was characterized by a serum 25(OH)D concentration of ≤75 nmol/L [21]; and vitamin D deficiency was indicated by a 25(OH)D level of ≤50 nmol/L [22] (to convert to ng/mL, divide by 2.496).

### 2.4. Statistical Analysis

The characteristics of participants were presented using frequencies and percentages or means ± standard deviations, as applicable. Initially, comparisons between participants were conducted by dividing them into two groups based on serum 25(OH)D concentration (i.e., either ≤50 nmol/L versus >50 nmol/L or ≤75 nmol/L versus >75 nmol/L). Quantitative variables were analyzed using Student’s *t*-test or Mann–Whitney–Wilcoxon test, depending on the normal distribution assumption, while qualitative variables were assessed using the Chi-square test or the Fisher exact test, as appropriate. Subsequently, univariate logistic regressions were employed to explore the relationships between clinical characteristics of participants (independent variables: each individual item from the VDSD tool) and hypovitaminosis D (dependent variable). Separate models were constructed for each definition of hypovitaminosis D. Lastly, the metrological properties of the entire VDSD tool (i.e., combination algorithm) were scrutinized for the identification of hypovitaminosis D within this cohort of geriatric patients with COVID-19. The *p*-values < 0.05 were deemed significant. All statistical analyses were performed using SAS^®^, version 9.4 (SAS Institute Inc., Cary, NC, USA).

## 3. Results

Out of 133 eligible participants, 102 individuals (76.7%) with complete data, no use of vitamin D supplements, and meeting the selection criteria (mean ± SD, 85.0 ± 5.9 years; 47.1% women; 100% Caucasian) were ultimately included in the current analysis. The mean 25(OH)D concentration was 44.0 ± 23.4 nmol/L, with 87 participants (85.3%) experiencing vitamin D insufficiency and 63 (61.8%) having vitamin D deficiency. Significant differences between groups were observed, particularly in terms of the daily number of drugs taken, fear of falling, history of falls, and undernutrition (Table 1).

Table 2 indicates that only a few clinical variables were individually associated with hypovitaminosis D.

Finally, Table 3 presents the metrological properties of the VDSD combinatorial algorithm for identifying hypovitaminosis D in the entire sample of older adults with COVID-19. Overall, the model correctly classified patients with hypovitaminosis D regardless of the definition used. However, the Kappa coefficient suggested that the tool exhibited better classification for individuals with or without vitamin D deficiency (≤50 nmol/L). The optimal performance was observed in identifying vitamin D deficiency, with an area under the curve (AUC) of 0.81 on the receiver operating characteristic (ROC) curve. The odds ratio (OR) for vitamin D deficiency was 40.0 (95% confidence interval: 10.5–152.3), with ‘not combining variables’ as the reference. Cohen’s Kappa coefficient for the VDSD result was 0.65 (95%CI: 0.50–0.80) compared to the result of the blood test. The VDSD also demonstrated moderate efficiency in identifying vitamin D insufficiency (AUC = 0.57; Cohen’s Kappa = 0.18 (95%CI: −0.07; 0.45)).

## 4. Discussion

Our findings indicate that the 16-item VDSD combinatorial algorithm effectively identified vitamin D deficiency (≤50 nmol/L) in older adults with COVID-19. While the VDSD was also capable of identifying vitamin D insufficiency (≤75 nmol/L), its efficiency was comparatively lower.

These results align with prior studies in hospitalized geriatric patients, emphasizing the VDSD’s effectiveness in detecting vitamin D deficiency, particularly [15]. Despite individual clinical variables showing modest or no association with hypovitaminosis D (Table 2), the amalgamation of the 16 items using the VDSD algorithm proved effective in identifying vitamin D deficiency in older inpatients with COVID-19. Therefore, our study contributes additional evidence by reporting the VDSD’s effectiveness in the context of COVID-19. Notably, the sensitivity was markedly high (Table 3), suggesting that the VDSD could serve as a valuable screening tool for hypovitaminosis D in the early stages of COVID-19, guiding the decision for blood collection. In this regard, an online tool incorporating the VDSD algorithm is currently under development. Clinicians can input the 16 items of the VDSD and the software will promptly provide the probability of hypovitaminosis D, along with recommendations for blood tests and/or vitamin D supplements in patients with COVID-19.

The identification of vitamin D deficiency is particularly crucial in older adults with COVID-19, as recent literature indicates survival benefits following the correction of hypovitaminosis D with supplements. Regular vitamin D supplementation has been associated with improved survival in older adults, especially when initiated recently or in the early stages of COVID-19, suggesting the desirability of achieving a high 25(OH)D concentration during COVID-19 to enhance prognosis. This is supported by the COVIT-TRIAL study, a randomized clinical trial demonstrating improved 14-day survival in older participants receiving a high dose of vitamin D3 compared to those receiving a standard dose [13].

However, it is essential to exercise caution in supplementing high doses of vitamin D “on sight” from the diagnosis of COVID-19. High-dose supplementation, aimed at elevating 25(OH)D concentration to supraphysiological levels, may not be useful and could be toxic among individuals without hypovitaminosis D. This contrasts with lower doses, which aim to prevent hypovitaminosis D and maintain 25(OH)D concentration at physiological levels. Given the potential U-shaped or reverse J-shaped effect of vitamin D [23], associating both low and high 25(OH)D concentrations with adverse health events, it becomes crucial to ascertain the vitamin D status of older individuals before initiating any vitamin D supplementation during COVID-19.

Despite the originality of our research question and its relevance in clinical practice, our study’s strengths include standardized data collection from a single research center, inclusion of geriatric patients of both sexes, consideration of different hypovitaminosis D definitions, and the utilization of a sophisticated non-linear artificial intelligence model. Artificial neural networks, inspired by the human brain, offer advanced capabilities in machine learning and pattern recognition, making them particularly suitable for understanding multifactorial mechanisms such as hypovitaminosis D. This explains why, although individual variables exhibited limited associations with vitamin D status in our study (Table 2), their combination using the VDSD combinatorial algorithm effectively identified hypovitaminosis D (Table 2). Importantly, the removal of variables from the 16-item VDSD resulted in a significant loss of diagnostic efficiency for severe vitamin D deficiency.

While artificial neural networks can continuously learn and improve with additional data, it is conceivable that future iterations of the VDSD may allow for a reduction in the number of required items.

Nevertheless, some limitations should be acknowledged. Our study cohort comprised inpatients with COVID-19 admitted to a geriatric ward, likely representing individuals with a severe form of COVID-19 or more serious comorbidities and lower 25(OH)D concentrations than the broader older patient population. The sample size was relatively small and could not be predetermined. All tests were conducted within a relatively short period, precluding an assessment of any seasonality effect. Additionally, our findings should be interpreted considering the limitations of the 25(OH)D chemiluminescent immunoassay, with liquid chromatography–tandem mass spectrometry (LC-MS/MS) being the gold standard. However, the radioimmunoassay offers reasonable cost, satisfactory intra- and inter-rater reliability, and simultaneous measurement of 25(OH)D2 and 25(OH)D3.

## 5. Conclusions

In conclusion, the 16-item VDSD effectively detected hypovitaminosis D in unsupplemented geriatric patients with COVID-19, particularly those with vitamin D deficiency (≤50 nmol/L). This rapid and cost-effective screening tool has the potential to assist clinicians in making informed decisions about supplementing their geriatric patients with COVID-19. Moving forward, it is important to explore the effectiveness of the VDSD in monitoring changes in vitamin D status following the initiation of vitamin D supplements.

## Figures and Tables

**Table 1:** Comparison of patients’ characteristics according to vitamin D status (*n* = 102).

Clinical Characteristics	Cohort of Older Adults with COVID-19						
	Whole Sample (*n* = 102)	Serum 25(OH)D Concentration, nmol/L					
		Vitamin D Deficiency ≤50 nmol/L (*n* = 63)	>50 nmol/L (*n* = 39)	*p*-Value *	Vitamin D Insufficiency ≤75 nmol/L (*n* = 87)	>75 nmol/L (*n* = 15)	*p*-Value *
**Item 1—Female sex**	48 (47.1)	28 (44.4)	20 (51.3)	0.501	42 (48.3)	6 (40.0)	0.553
**Item 2—Age, years (mean** **±** **SD)**	85.0 ± 5.9	85.2 ± 6.0	84.6 ± 6.0	0.662	85.3 ± 5.7	83.1 ± 7.1	0.196
**Item 3—Number of drugs daily taken (mean** **±** **SD)**	6.9 ± 3.7	6.9 ± 3.7	6.9 ± 3.6	0.860	7.3 ± 3.7	5.1 ± 3.1	**0.016**
**Item 4—Body mass index, kg/m^2^ (mean** **±** **SD)**	26.9 ± 4.6	27.5 ± 4.7	26.0 ± 4.3	0.121	27.2 ± 4.7	25.2 ± 3.8	0.109
**Item 5—Use walking aids**	60 (58.8)	37 (58.7)	23 (59.0)	0.981	52 (59.8)	8 (53.3)	0.640
**Item 6—Use psychoactive drugs**	43 (42.2)	23 (36.5)	20 (51.3)	0.142	40 (46.0)	3 (20.0)	0.060
**Item 7—Wearing glasses**	98 (96.1)	60 (95.2)	38 (97.5)	1.000	84 (96.6)	14 (93.3)	0.472
**Item 8—Sad mood**	25 (24.5)	13 (20.6)	12 (30.8)	0.248	21 (24.1)	4 (26.7)	1.000
**Item 9—Fear of falling**	48 (47.1)	36 (57.1)	12 (30.8)	**0.010**	44 (50.6)	4 (26.7)	0.087
**Item 10—History of falls**	39 (38.2)	30 (47.6)	9 (23.1)	**0.013**	35 (40.2)	4 (26.7)	0.318
**Item 11—Cognitive disorders**	50 (49.0)	30 (47.6)	20 (51.3)	0.719	44 (50.6)	6 (40.0)	0.449
**Item 12—Undernutrition**	13 (12.8)	6 (9.5)	7 (18.0)	0.236	8 (9.2)	5 (33.3)	**0.022**
**Item 13—Polymorbidity**	51 (50.0)	33 (52.4)	18 (46.2)	0.541	43 (49.4)	8 (53.3)	0.780
**Item 14—History of vertebral fractures**	7 (6.9)	4 (6.4)	3 (7.7)	1.000	6 (6.9)	1 (6.7)	1.000
**Item 15—Living alone**	31 (30.4)	18 (28.6)	13 (33.3)	0.611	26 (29.9)	5 (33.3)	0.769
**Item 16—Use anti-osteoporotic drugs**	12 (11.8)	10 (15.9)	2 (5.1)	0.124	12 (13.8)	0 (0.0)	0.205
**25-hydroxyvitamin D, nmol/L (mean** **±** **SD)**	44.0 ± 23.4	28.0 ± 11.3	69.8 ± 11.4	**<0.001**	37.4 ± 18.4	82.1 ± 4.4	**<0.001**

Data presented as *n* (%) where applicable; SD: standard deviation; *: based on *t*-test or Chi-square test, as appropriate; *p*-value significant (i.e., <0.05) indicated in bold.

**Table 2:** Univariate logistic regression models examining the cross-sectional associations between patients’ clinical characteristics and hypovitaminosis D (*n* = 102).

	Hypovitaminosis D					
	Vitamin D Deficiency 25(OH)D ≤ 50 nmol/L			Vitamin D Insufficiency 25(OH)D ≤ 75 nmol/L		
	OR	[95%CI]	*p*-Value	OR	[95%CI]	*p*-Value
**Item 1—Female sex**	0.76	0.34–1.69	0.502	1.40	0.46–4.27	0.554
**Item 2—Age, years**	1.02	0.95–1.09	0.658	1.07	0.97–1.18	0.197
**Item 3—Number of drugs daily taken**	1.00	0.90–1.12	0.986	1.21	1.01–1.43	**0.035**
**Item 4—Body mass index, kg/m^2^**	1.08	0.98–1.18	0.124	1.11	0.97–1.27	0.120
**Item 5—Use walking aids**	0.99	0.44–2.23	0.981	1.30	0.43–3.91	0.641
**Item 6—Use psychoactive drugs**	0.55	0.24–1.23	0.144	3.40	0.90–12.92	0.072
**Item 7—Wearing glasses**	0.53	0.05–5.25	0.585	2.00	0.19–20.61	0.560
**Item 8—Sad mood**	0.59	0.24–1.46	0.250	0.88	0.25–3.04	0.833
**Item 9—Fear of falling**	3.00	1.29–6.97	**0.011**	2.81	0.83–9.52	0.096
**Item 10—History of falls**	3.03	1.24–7.41	**0.015**	1.85	0.55–6.28	0.323
**Item 11—Cognitive disorders**	0.86	0.39–1.92	0.719	1.54	0.50–4.68	0.452
**Item 12—Undernutrition**	0.48	0.15–1.55	0.222	0.20	0.06–0.74	**0.016**
**Item 13—Polymorbidity**	1.28	0.58–2.86	0.541	0.86	0.29–2.56	0.780
**Item 14—History of vertebral fractures**	0.81	0.17–3.85	0.795	1.04	0.12–9.28	0.974
**Item 15—Living alone**	0.80	0.34–1.89	0.612	0.85	0.27–2.74	0.789
**Item 16—Use anti-osteoporotic drugs**	3.49	0.72–16.86	0.120	na	na	na
**Prediction of vitamin D deficiency** **according to VDSD tool**	40.00	10.51–152.31	**<0.001**	-	-	-
**Prediction of vitamin D insufficiency** **according to VDSD tool**	-	-	-	4.10	0.87–19.4	0.075

CI: confidence interval; 25(OH)D: 25-hydroxyvitamin D; OR: odds ratio; na: not applicable due to almost complete separation of data; *p*-value significant (i.e., <0.05) indicated in bold.

**Table 3:** Metrological properties of the VDSD tool for the identification of hypovitaminosis D according to the different definitions of hypovitaminosis D (*n* = 102).

Hypovitaminosis D	True Positive	False Positive	True Negative	False Negative	Sensitivity%	Specificity%	Positive Predictive Value	Negative Predictive Value	Accuracy %	Cohen’s Kappa (95%CI)
**Vitamin D insufficiency ≤ 75 nmol/L**	82	12	3	5	94.3	20.0	87.2	37.5	83.3	0.18 (−0.07; 0.45)
**Vitamin D deficiency ≤ 50 nmol/L**	60	13	26	3	95.2	66.7	82.2	89.7	84.3	0.65 (0.50; 0.80)

## Data Availability

Patient level data are freely available at drci@chu-angers.fr to qualifying researchers registered with an appropriate institution and who submit a proposal with a valuable research question. There is no personal identification risk within this anonymized raw data, which is available after notification and authorization of the competent authorities.
